# Forensically informative nucleotide sequencing (FINS) for species and subspecies of genus *Prionailurus* (Mammalia: Carnivora: Felidae) through mitochondrial genes (12SrRNA and cytochrome b) by using old taxidermy samples

**DOI:** 10.1080/23802359.2018.1462115

**Published:** 2018-05-17

**Authors:** Archana Bahuguna

**Affiliations:** Molecular Systematic Laboratory, Northern Regional Centre, Zoological Survey of India, Dehradun, India

**Keywords:** FINS, *Prionailurus*, old taxidermy samples, maximum likelihood, neighbour joining

## Abstract

Genus *Prionailurus* comprises four species, i.e. Leopard cat, Rusty spotted cat, Fishing cat, Flat-headed cat, listed under IUCN as threatened species except *P. bengalensis.* In U.S.A., *P. bengalensis* is listed as Endangered. Subspecies of *P. bengalensis*, i.e. *iriomotensis* is listed as Critically Endangered under IUCN since 2008. The present study describes the use of two markers 12SrRNA and cytochrome b genes to differentiate the three species and three subspecies of *Prionailurus.* Old taxidermy samples (three skin samples) of *P. viverrinus* and *P.b. horsfieldi* were used from India for the study. The study done by using DNAsp v5, MEGA 6.0, and Network 5.0.0.1, proved that both gene markers are useful for differentiating the species and subspecies of *Prionailurus*. This study is also the first study to present forensically informative nucleotide sequence (FINS) for three species and three subspecies of *Prionailurus*.

## Introduction

The genus *Prionailurus* comprises of species Leopard cat (*Prionailurus bengalensis*), Rusty spotted cat (*Prionailurus rubiginosus*), Fishing cat (*Prionailurus viverrinus*), and Flat-headed cat (*Prionailurus planiceps*). Leopard cats are the most widely distributed Asian small cats (Nowell and Jackson 1996; Sunquist and Sunquist 2002). Their range with subspecies bengalensis, *javanensis, sumatranus, chinensis, horsfieldi euptilurus/euptilura, borneoensis, trevelyani*, *alleni, iriomotensis, heaneyi*, and *rabori* extends from the Amur region in the Russian Far East over the Korean Peninsula, China, Indochina, the Indian Subcontinent, to the West in northern Pakistan, and to the south in the Philippines and the Sunda islands of Indonesia (Ross et al. 2010). *P.b. horsfieldi* ranges in Kashmir, Punjab, Kumaon, Nepal, Sikkim, and Bhutan (Vella et al. 2002); Amur leopard cat *P.b. euptilurus/euptilura* is distributed in eastern Siberia, Manchuria, and Korea (Vella et al. 2002) and on the Tsushima Island (Werdelin and Olsson 2008). Iriomote cat *P.b. iriomotensis* is found exclusively on the tiny island of Iriomote in the Japanese Archipelago (Imaizumi 1967).

Leopard cat, Fishing cat (Endangered by IUCN, A2cd +4cd ver 3.1), Flat headed cat (Endangered C1 ver 3.1.) are commercially traded internationally for the fur trade and also killed in retribution (Nowell and Jackson 1996; Duckworth et al. 2009; Ross et al. 2010, http://www.iucnredlist.org).

Mitochondrial 12S rRNA and cytochrome b genes have been applied to understand the interspecies and intraspecies genetic diversity. In the present study, two skin samples were used belonging to *P.b. horsfieldi* and *P. viverrinus* for amplification of genes, i.e. 12SrRNA and cytochrome b and of other species and subspecies of the genus *Prionailurus* were used from NCBI. Since the gene sequences (12SrRNA and cytochrome b) of only two subspecies, i.e. *P.b. iriomotensis* and *P.b. euptilurus* are available at NCBI thus the present study is confined to three subspecies of *P. bengalensis*, i.e. *P.b. horsfieldi, P.b. euptilurus*, and *P.b. iriomotensis.* This study is a first attempt of molecular characterization of three species out of four known species of genus *Prionailurus*, i.e. *P. bengalensis*, *P. viverrinus*, and *P. planiceps* and (as no nucleotide database is available for *P. rubiginosus*) and four subspecies *P.b. iriomotensis* and *P.b. euptilurus* of *P. bengalensis horsfieldi* and *P.b. bengalensis.*

This study is useful in wildlife forensic for species identification and enforcement of law and regulations by providing strong scientific proofs and for their status based on forensically informative nucleotide sequencing (FINS) (Bartlett and Davidson 1992). The technique combines DNA sequencing and phylogenetic analysis. The author discussed the genetic difference between three subspecies of *P.b. horsfieldi, P.b. iriomotensis*, and *P.b. euptilurus* hence the origin of the seized wildlife parts and products.

## Materials and methods

### Sample collection

In this study, taxidermy skin samples were used. These taxidermy samples (0.5 cm × 0.5 cm) were collected from Mammals section of Zoological Survey of India, Kolkata (Table 1). Additionally, 12s rRNA and cytochrome b available sequences of small cats of genus *Prionailurus* were obtained from GenBank (Table 1).

**Table 1.** Sample details.

Species	*P.b. iriomotensis*	*P.b. euptilura*	*P.b. horsfieldi*	*P. bengalensis*	*P. viverrinus*	*P. planiceps*
12srRNA gene						
S	From NCBI	From NCBI	Menam, Sikkim, India, DOC 20.05.1990	From NCBI	Satpura (Orissa, District Puri), India, DOC16.03.14	From NCBI
SID	Pbi	Pbe	Pbh Reg no. 24292, ZSI collection, C: S.Chattopadhaya	Pb	Pv Reg no. 10022, ZSI collection; C: W.Rutledge	Pp
Accession no.	D28888.1	JF693229.1	KM093870.1	D28890.1, D28889.1	KM093869.1	Sequence not available
Cyt b gene						
S	From NCBI	From NCBI	Sequence not available	From NCBI	From NCBI	From NCBI
SID	Pbi	Pbe	Pbh		Pv	Pp
	AB210228	FJ788107	NA	KF754931.1	AB210240, AB210239.1	FJ594958

S: sample locality with DOC (date of collection); C: collector; s: sample source; SID: sample ID.

### DNA isolation, PCR amplification, and sequencing

After washing with MilliQ water and cleaning, the skin samples were hydrated before digestion by incubating the dried skin sample for 24 h in 1 ml TE solution (Tris 10 mM and EDTA 1 mM, pH 7.6) (Barros and Morgante 2007). After 24 h of hydration, the DNA was isolated from skin sample using HiPur A^TM^ Forensic Sample Genomic DNA Purification Kit (HIMEDIA).

12s rRNA sequences were amplified using a set of primer pair, L1091 and H1478, and a primer set of L14841 and H15149 was used to amplify cytochrome b gene (Kocher et al. 1989). The PCR reaction was performed in Q-cycler, Quanta Biotech (Surrey, UK), in a total volume of 25 µl of reaction mixture (10× PCR-with MgCl_2_, 2.5 µl; 10 mM dNTP’s, 2.5 µl; 5 pmol primer, 0.45 µl each; 15 ng of DNA template; 1.5 U Taq enzyme). Polymerase chain reaction consisted of initial denaturation of 94 °C for four minutes and each cycle of denaturation for 1 min at 94 °C, hybridization for 1 min at 55 °C (50 °C for cytochrome b) and extension for 1 min at 72 °C followed by final elongation for 10 min at 72 °C. The cycle was repeated for 35 times. The PCR products were sequenced using ABI's AmpliTaq FS dye terminator cycle sequencing chemistry on an automated ABI 3100 Genetic Analyser. All experiments were performed in a PCR workstation (Bangalore GeNei^TM^). Negative controls were used in all DNA extraction and PCR amplification to control for potential contamination. 12SrRNA gene and cytochrome b gene sequences thus generated are submitted to NCBI after conducting sequence alignment by Bioedit and by checking their similarity with species of genus *Prionailurus*.

### Data analysis

Sequences were visualized and edited using Chromas 1.6 (Technelysium Pty Ltd., South Brisbane, Australia). To crosscheck, quarry sequences were compared using GenBank BLAST (http://www.ncbi.nlm.nih.gov/BLAST). CLUSTAL W was used to compare DNA sequence data implemented in BioEdit v 7.0.9.0 software (Hall 1990) with outgroup *Canis lupus lupus* (AM711902.1). All sequences were proof read and analysed by using MEGA6.0 (Tamura et al. 2011) and were aligned by using ClustalW (Thompson et al. 1994). MEGA6.0 was used for finding the conserved, variable, parsimony informative and singleton sites as well as for phylogeny construction. Two methods were used for phylogeny construction: (i) maximum likelihood and (ii) neighbour joining with Kimura 2 Parameter (Pevsner 2009). All trees were subjected to bootstrap analysis with 1000 replicates to get bootstrap value support. The complete mitochondrial genome of *Cunis lupus lupus* was retrieved from NCBI database to be used as outgroup.

## Results and discussion

12Sr RNA gene was amplified from extracted DNA of *P. viverrinus, P.b. bengalensis*, and *P.b. horsfieldi.* After sequencing, the unambiguous lengths of 12S rRNA (ca. 377 bp) were obtained and the same was tried for cytochrome b. The BLAST result indicated that the obtained sequences matched 99–100% with their respective species. In 12srRNA with 377 bp, the author observed five haplotypes, 312 conserved sites, 59 variable sites, 17 parsimony informative sites, and 41 singleton sites and in cytochrome b with 1100 bp, the author observed seven haplotypes; 816 conserved sites, 284 variable sites, 84 parsimony informative sites, and 200 singleton sites.

### Species specific sites in 12S rRNA gene

In relation to the complete mitochondrial genome of *Canis lupus lupus* out of 1100 bp of 12SrRNA gene, there were 320, 320, 319, 321, and 301 conserved sites, 44, 44, 44, 43, and 58 variable sites and 49, 49, 47, 48, and 63 parsimony informative sites were observed in *P.b. iriomotensis, P.b. euptilurus, P. bengalensis*, *P.b. horsfieldi*, and *P. viverrinus*, respectively. Six singleton, one singleton, and 26 singleton sites were observed in *P. bengalensis* (D28889.1), *P. bengalensis* (D28890.1), and *P. viverrinus* (KM093869.1), respectively, and for *P.b. iriomotensis*, *P.b. euptilurus*, and *P. bengalensis horsfieldi* it was noted to be zero. The nucleotide composition of the entire sequence region of 12S rRNA gene (377 bp) is A 36.25%, T/U 24.39%, C 21.77%, and G 17.59%.

**Figure 1.**
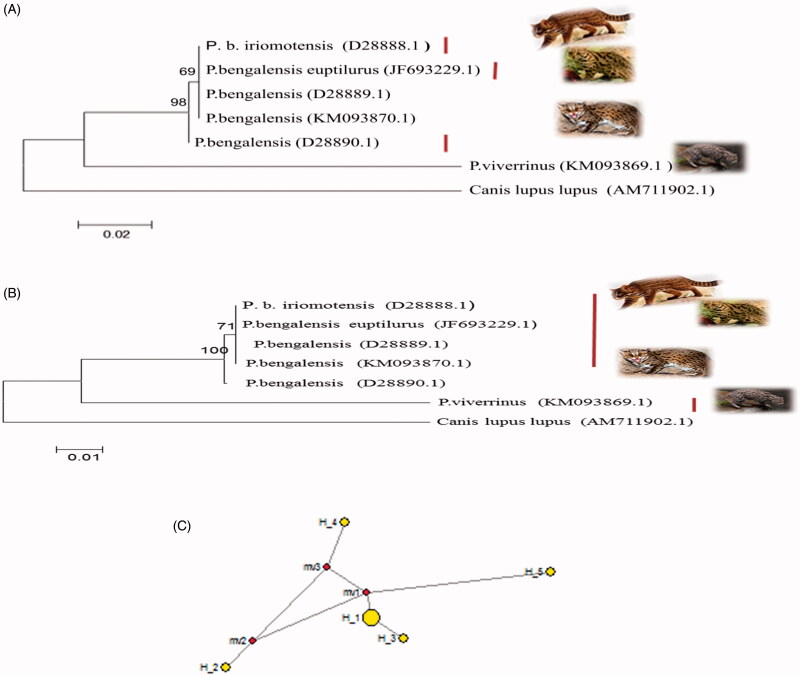
12SrRNA based ML tree topology (A) and NJ tree topology (B) of species and subspecies of *Prionailurus*. The evolutionary distances were computed using the Kimura two-parameter method and are in the units of the number of base substitutions per site. The analysis involved seven nucleotide sequences including outgroup *Canis lupus lupus*. Median-joining network by using 12SrRNA gene of genus *Prionailurus* indicating four haplotypes of species and subspecies of *Prionailurus*. Haplotype 5 belongs to outgroup (C).

Concurrent evolutionary trees were obtained by using MEGA 6 with maximum likelihood and neighbour joining algorithms (Figure 1(A,B)), where *P.b. iriomotensis* and *P.b. euptilurus*, along with *P. bengalensis* (D28889) and *P. bengalensis* (D28890) and *P.b. horsfieldi* formed one clade. However, by using DnaSPv5 and network analysis by using NETWORK software indicated that haplotype 1 belongs to *P. viverrinus* and haplotype 2 is shared by *P.b. iriomotensis, P.b. euptilurus*, and *P. bengalensis* (D28889) indicating the common origin. Haplotype 3 belongs to *P. bengalensis* (D28890.1 with unknown subspecies) and haplotype 4 belongs to *P.b. horsfieldi* (Figure 1(C)).

### Cytochrome b gene analysis

Genetic analysis was done by MEGA 6 with seven sequences, ca. 1100 bp with *Canis lupus lupus* as outgroup inferred 284 variable/polymorphic (segregating) sites and seven haplotypes.

**Figure 2.**
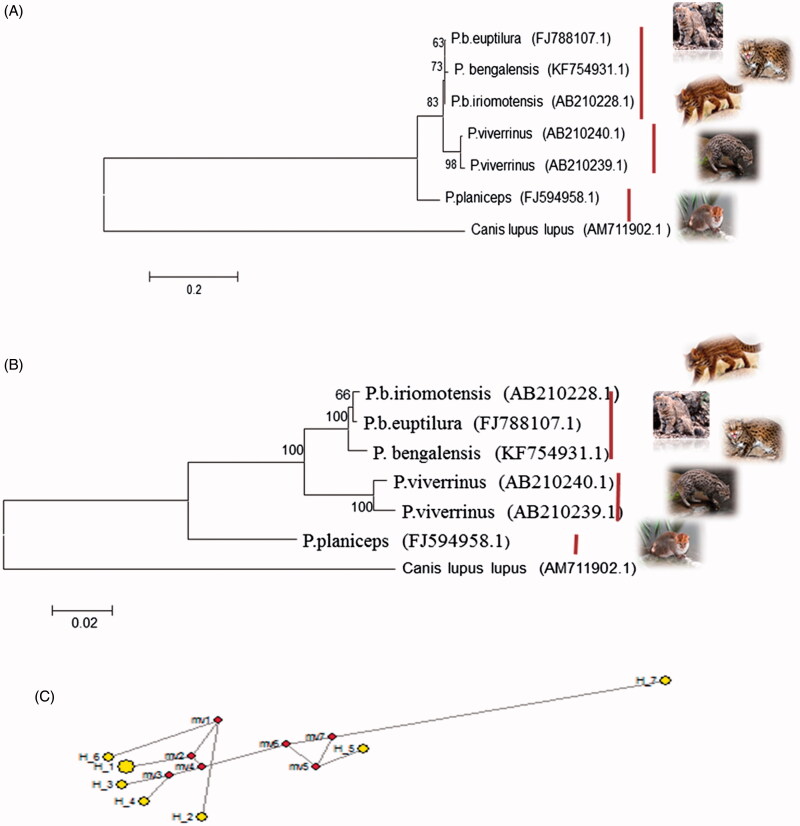
Cytochrome b based ML tree topology (A) and NJ tree topology (B) of species and subspecies of *Prionailurus*. The evolutionary distances were computed using the Kimura two-parameter method and are in the units of the number of base substitutions per site. The analysis involved seven nucleotide sequences. Median-joining network (C) by using cytochrome b gene of genus *Prionailurus* indicating six haplotypes of species and subspecies of *Prionailurus*. Haplotype 7 belongs to outgroup.

In relation to the complete mitochondrial genome of *Canis lupus lupus* (AM711902.1) out of 1100 bp cytochrome b sequence as determined by MEGA 6, there were 868, 870, 865, 886, and 867 conserved sites; 232, 230, 233, 214, and 233 variable sites and 223, 231, 244, 216, and 232 parsimony informative sites in *P.b. irimotensis*, *P.b. euptilurus*, *P. viverrinus*, *P. planiceps*, and *P. bengalensis*, respectively. Singleton sites were 0 in *P.b. irimotensis*, two in *P.b. euptilurus*, 16 in *P. viverrinus*, 17 in *P. planiceps*, and two in *P. bengalensis*. These sites can be used to differentiate the species and subspecies of *Prionailurus*. Six haplotypes were present. Hap 1 for *P.b. iriomotensis*, Hap 2 for *P.b. euptilurus*, Hap3 and Hap 4 belongs to *P. viverrinus*, Hap 5 belongs to *P. planiceps* (Table 2 and Figure 2(C)). The nucleotide compositions of the entire sequenced region of Cytb (1100 bp) are A 28.5%, T/U 27.1%, C 31.1%, and G 13.4%.

**Table 2.** Observed DNA diversity in 12Sr RNA and cytochrome b gene.

Species	*P.b. iriomotensis*	*P.b. euptilura*	*P.b. horsfieldi*	*P. bengalensis*	*P. viverrinus*	*P. planiceps*
12Sr RNA						
H	Hap2	Hap2	Hap 4	Hap 2 and Hap 3 *P. bengalensis*	Hap1	Not available
V	44	44	43	44	58	Not available
S	0	0	0	6 and 1	26	Not available
Cyt b						
H	1	1	Sequence not available	1	2	1
V	232	230	–	233	233	214
S	0	2	–	2	16	17

H: haplotype sites; V: variable sites; S: singleton sites.

Phylogenetic study by using maximum likelihood and neighbour joining algorithms (MEGA 6) produced congruent trees (Figure 2(A,B)) with three clades where all subspecies sequences of *P. bengalensis*, i.e. *irimotensis*, *euptilurus*, and *bengalensis* form one clade, whereas *P. viverrinus* and *P. planiceps* were present in clade B and clade C, respectively, with 100% bootstrap value.

Among the species and subspecies examined in the present study, it was noted that the iriomote cat is a subspecies of *P. bengalensis* and not a distinct species based on genetic and evolutionary analysis and *P.b. iriomotensis* and *P.b. euptilurus*, i.e. Amur leopard cat, shared similar haplotype of 12SrRNA, but *P.b. horsfieldi* has distinct haplotype for 12SrRNA gene.

Amur Leopard Cat *P.b. euptilurus* was earlier proposed as a distinct species based on morphological differences from southeast Asian specimens, but Chinese specimens have been shown to be similar to those from southeast Asia (Wozencraft 2005). In a molecular study done by Masuda and Yoshida (1995), a clear distinction was noted between northern populations from Tsushima, Korea, Siberia, China and Taiwan and south east Asian populations. If these genetic differences indicate a specific distinction, *P.b. euptilurus* may yet be a valid species. The Iriomote cat (*P.b. iriomotensis*) from Japan's Iriomote island was also originally described as a distinct species based on morphology (Imaizumi 1967), but based on mt DNA analysis, it is now considered a subspecies of Leopard Cat (Masuda and Yoshida 1995; Johnson et al. 1999; Wozencraft 2005). The present study also reveals that the Iriomote cat is a subspecies of *P. bengalensis* and genetically similar to *P.b. euptilurus*. It has been classified as Critically Endangered by IUCN since 2008, as the population size is fewer than 250 thus there is need to apply conservation measures. Recent genetic analysis (Luo et al. 2014) suggests species-level distinction between the Indochinese and Sundaic populations of the Leopard Cat. DNA sequence, 12SrRNA, and cytochrome b comparisons were used by Janczewski et al. (1995) to infer phylogenetic relationships among 17 Felid species and supports the use of the two genes for genetically differentiating the species and subspecies for small cats. According to Janczewski et al. (1995), cytochrome b sequences appear to accumulate differences rapidly and then stabilize, whereas 12SrRNA differences with lower number of changes per variable sites seem to accumulate more gradually but steadily.

## Conclusions

Cytochrome b is potentially good to determine the genetic difference between the two subspecies of *P. bengalensis*, i.e. *iriomotensis* and *euptilurus* and species of *Prionailurus* hence in providing the strong scientific proof for identification of the subspecies. However, 12SrRNA is effective to differentiate *P. bengalensis horsfieldi* from *P.b. iriomotensis* and *P.b. euptilurus*.
